# Additional Evidence of the Trypanocidal Action of (−)-Elatol on Amastigote Forms through the Involvement of Reactive Oxygen Species

**DOI:** 10.3390/md12094973

**Published:** 2014-09-25

**Authors:** Vânia Cristina Desoti, Danielle Lazarin-Bidóia, Daniela Bueno Sudatti, Renato Crespo Pereira, Tania Ueda-Nakamura, Celso Vataru Nakamura, Sueli de Oliveira Silva

**Affiliations:** 1 Postgraduate Program in Pharmaceutical Sciences, State University of Maringa, Colombo Avenue 5790, Maringa, Parana CEP 87020-900, Brazil; E-Mails: vaniadesoti@gmail.com (V.C.D.); dani.lazarin@bol.com.br (D.L.-B.); tunakamura@uem.br (T.U.-N.); cvnakamura@gmail.com (C.V.N.); 2 Department of Marine Biology, Federal Fluminense University, P.O. Box 100644, Niteroi, Rio de Janeiro CEP 24001-970, Brazil; E-Mails: dbsudatti@gmail.com (D.B.S.); rcrespo@id.uff.br (R.C.P.); 3 Department of Basic Health Sciences, State University of Maringa, Colombo Avenue 5790, Maringa, Parana CEP 87020-900, Brazil

**Keywords:** (−)-elatol, *Laurencia dendroidea*, *Trypanosoma cruzi*, Chagas’ disease, reactive oxygen species

## Abstract

Chagas’ disease, a vector-transmitted infectious disease, is caused by the protozoa parasite *Trypanosoma cruzi*. Drugs that are currently available for the treatment of this disease are unsatisfactory, making the search for new chemotherapeutic agents a priority. We recently described the trypanocidal action of (−)-elatol, extracted from the macroalga *Laurencia dendroidea*. However, nothing has been described about the mechanism of action of this compound on amastigotes that are involved in the chronic phase of Chagas’ disease. The goal of the present study was to evaluate the effect of (−)-elatol on the formation of superoxide anions (O_2_^•−^), DNA fragmentation, and autophagy in amastigotes of *T. cruzi* to elucidate the possible mechanism of the trypanocidal action of (−)-elatol. Treatment of the amastigotes with (−)-elatol increased the formation of O_2_^•−^ at all concentrations of (−)-elatol assayed compared with untreated parasites. Increased fluorescence was observed in parasites treated with (−)-elatol, indicating DNA fragmentation and the formation of autophagic compartments. The results suggest that the trypanocidal action of (−)-elatol might involve the induction of the autophagic and apoptotic death pathways triggered by an imbalance of the parasite’s redox metabolism.

## 1. Introduction

Chagas’ disease is a vector-transmitted infectious disease that is caused by the protozoa parasite *Trypanosoma cruzi*. It affects approximately 10 million individuals in worldwide [1]. This disease is appearing in non-endemic areas, because of immigration, through non-vectorial transmission mechanisms, such as congenital, blood transfusion and organs donation [2]. In turn, it is appearing in endemic countries mainly through of foodborne transmission, which have attracted great attention [3]. The available therapies for this infection are based on two nitroheterocyclics, nifurtimox and benznidazole, that are unsatisfactory because they present low efficacy, with cure rates of 60% in the acute phase and only 10%–20% in the chronic phase of the disease [4]. Additionally, serious toxic side effects are associated with nifurtimox and benznidazole [4,5] with prolonged treatment [6]. Thus, the search for new chemotherapeutic agents is a priority.

**Figure 1:**
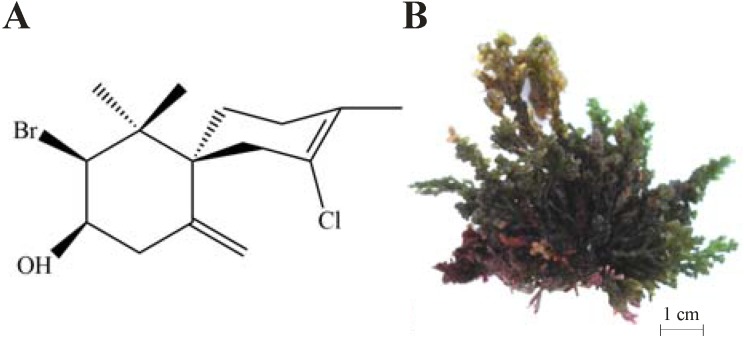
Chemical structure of (−)-elatol (**A**), the halogenated sesquiterpene extracted from the red macroalga *Laurencia dendroidea* (**B**).

Numerous compounds have been studied for the treatment of Chagas’ disease [7]. For example, the literature presents studies of extracts and pure compounds obtained from marine algae with trypanocidal activity [8,9,10], but little is known about the biochemical alterations induced by them. We recently described the trypanocidal mechanism of action of (−)-elatol, a halogenated sesquiterpene extracted from the red macroalga *Laurencia dendroidea* (Figure 1), collected on the Brazilian coast, on trypomastigote forms of *T. cruzi* [11]. However, nothing has been described about the mechanism of action of this compound on amastigote forms that are involved in the chronic phase of Chagas’ disease. Both forms, trypomastigotes (nonreplicative) and amastigotes (replicative), are infective forms found in the vertebrate host [12].

Considering the trypanocidal activity of (−)-elatol and lack of information on the mechanism of action in amastigote forms of *T. cruzi*, the present study provides data on the probable mode of trypanocidal action. Based on the ultrastructural alterations observed in intracellular amastigotes treated with (−)-elatol [10] and autophagic compartments and DNA fragmentation observed herein, we conclude that amastigotes treated with (−)-elatol died through autophagic and apoptotic processes. Our results provide further insights into the mechanism of action of (−)-elatol, strongly suggesting that (−)-elatol may be an effective treatment for Chagas’ disease with remarkable trypanocidal action against the amastigote forms of *T. cruzi*.

## 2. Results and Discussion

Natural products have proven to be valuable sources of new therapeutic agents that act against infectious and noninfectious diseases by providing an alternative to conventional treatments [7]. However, few studies of marine natural products have been conducted, despite the fact that marine algae have been used in traditional remedies in many Asian countries [13]. These natural products hold great promise. For example, bioactive compounds [14,15,16], such as the halogenated sesquiterpene (−)-elatol from *Laurencia dendroidea*, have significant biological activities. Some studies have demonstrated the chemotherapeutic properties of this compound, including antibacterial [17,18,19] and antiprotozoal activity [10,20], especially in the amastigote forms of *T. cruzi* [10]. Therefore, we sought to delineate the putative mechanism of action of (−)-elatol in amastigote forms.

**Figure 2:**
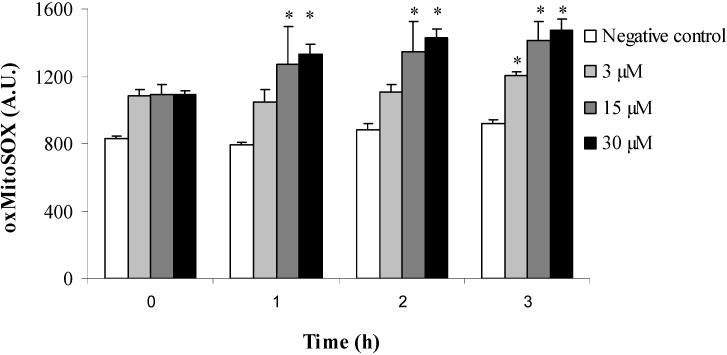
Mitochondrial O_2_^•−^ production in amastigote forms of *Trypanosoma cruzi* treated with 3, 15, and 30 μM (−)-elatol for up to 3 h using the fluorescent probe MitoSOX. At the indicated times, amastigotes were used to fluorimetrically measure oxidized MitoSOX (oxMitoSOX). The results are expressed in arbitrary units (mean ± SD of at least three independent experiments). * *p* ≤ 0.05, significant differences relative to the negative control (untreated cells; two-way analysis of variance followed by Tukey *post hoc* test).

Based on our previous results obtained with trypomastigotes treated with (−)-elatol [11], we evaluated the production of superoxide anions (O_2_^•−^), a reactive species of oxygen (ROS). The production of this radical was measured using a highly sensitive fluorimetric assay in mitochondria of the amastigote forms of *T. cruzi* treated with (−)-elatol. As shown in Figure 2, (−)-elatol significantly increased O_2_^•−^ production at all concentrations of (−)-elatol tested over 3 h compared with untreated cells. A 60% increase was observed in higher concentration compared with the negative control. The positive control with antimicyn A also increased mitochondrial O_2_^•−^ production (data not shown).

**Figure 3:**
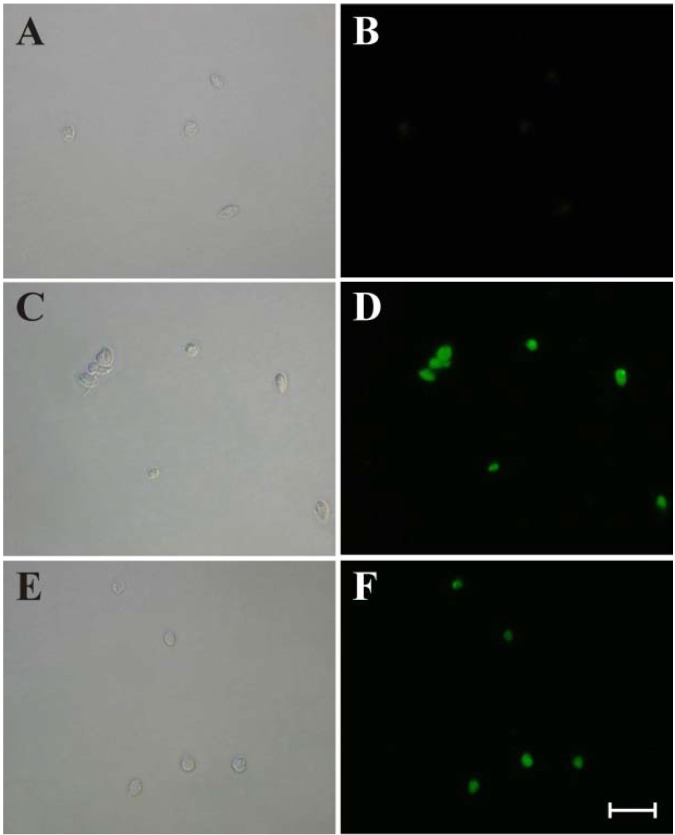
DNA fragmentation in amastigote forms of *Trypanosoma cruzi* treated with (−)-elatol for 24 h using TUNEL assay. The gray column is differential interference contrast (DIC), and the black column is fluorescence. Untreated amastigote forms (**A** and **B**). Amastigote forms treated with 1.5 μM (−)-elatol (**C** and **D**). Amastigote forms treated with 3 μM (−)-elatol (**E** and **F**). Scale bar = 10 μM.

The increase in O_2_^•−^ production, induced by (−)-elatol, might induce radical reactions triggering a cascade of damage, such as a break in DNA that is a hallmark of apoptotic death [21]. The apoptotic process is associated with signaling cascades involving mitochondria (intrinsic pathway) or death receptors (extrinsic pathway) [22]. In both pathways ROS can act as signaling molecules [23]. As shown in Figure 3, bright fluorescence, indicating DNA fragmentation, was observed in amastigote forms treated with 1.5 and 3 μM (−)-elatol for 24 h and subjected to the TUNEL assay (Figure 3D,F, respectively). The untreated control was TUNEL-negative (Figure 3B). Additionally, bright fluorescence was observed with actinomycin D, a known inducer of apoptosis (data not shown).

**Figure 4:**
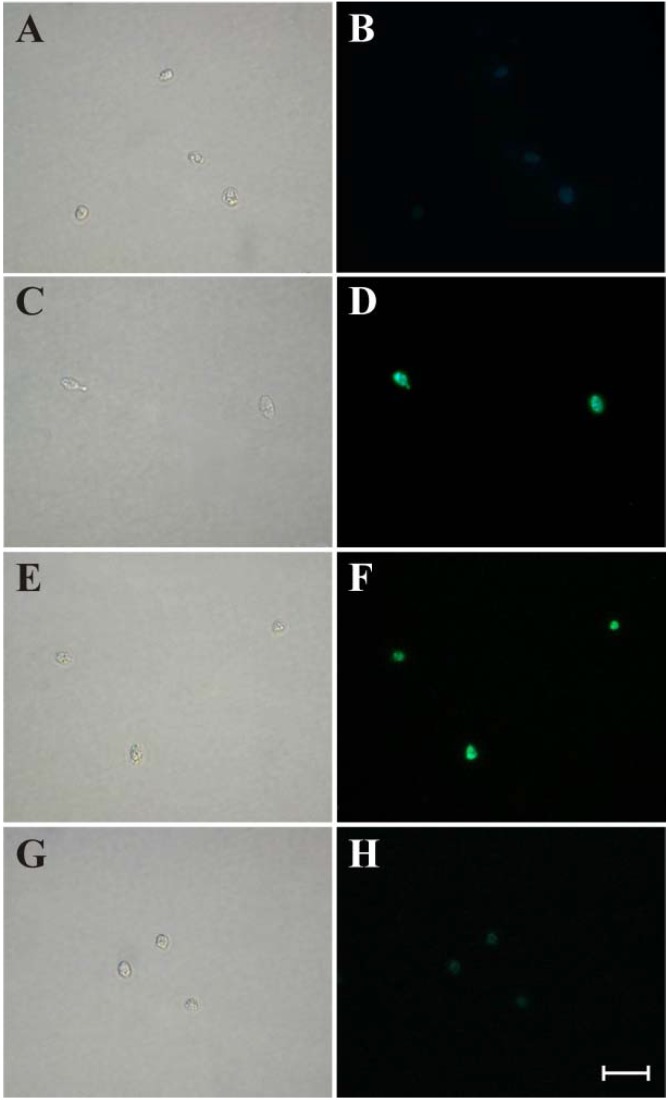
Autophagic compartments in amastigote forms of *Trypanosoma cruzi* treated with (−)-elatol for 24 h and stained with monodansylcadaverine. The gray column is differential interference contrast (DIC), and the black column is fluorescence. Untreated amastigote forms (**A** and **B**). Amastigote forms treated with 1.5 μM (−)-elatol (**C** and **D**). Amastigote forms treated with 3 μM (−)-elatol (**E** and **F**). Amastigote forms treated with 3 μM (−)-elatol + 500 nM wortmannin (**G** and **H**). Scale bar = 10 μM.

Based on our previous work that showed extensive vacuolization in the amastigote and trypomastigote forms of *T. cruzi* treated with (−)-elatol, demonstrated by transmission electron microscopy and fluorescence microscopy, respectively [10,11], we assessed whether autophagy is an alternative pathway to cell death induced by (−)-elatol in amastigote forms. Autophagy is a mechanism that involves degradation of unnecessary or dysfunctional cellular molecules through the actions of lysosomes/vacuole [24]. The cellular damage might be result from the high levels of ROS that can oxidize macromolecules [25]. Thus, we evaluated autophagy in amastigotes treated with (−)-elatol and stained with monodansylcadaverine, a fluorescent marker that accumulates in autophagic vacuoles [26]. Figure 4 shows the presence of fluorescent, rounded structures in cells treated with (−)-elatol, indicating the formation of autophagic compartments (Figure 4D,F), in contrast to untreated cells (Figure 4B). This effect could be partially prevented in amastigotes that were pretreated with wortmannin (Figure 4H).

The results presented above indicate that (−)-elatol induces alterations that might be responsible for two types of cell death, apoptosis (demonstrated by DNA fragmentation; type I programmed cell death [PCD]) [27,28] and autophagy (demonstrated by the formation of autophagic vacuoles; type II PCD) [28], in amastigote forms of *T. cruzi*. Apoptosis has several classic characteristics, such as DNA fragmentation, which is one of the final steps in the apoptotic process [21,23]. Autophagy is characterized by an increase in cytoplasmic vacuolization [29,30]. Both cell death pathways have been well described for trypanosomatids, with significant mitochondrion and ROS participation [31,32]. However, independent of the pathway, (−)-elatol affected mitochondrial function by increasing mitochondrial O_2_^•−^ production. Reactive oxygen species might trigger biochemical alterations that lead to cell death. Interestingly, the evidence suggests that the transition from apoptosis or autophagy is associated with excessive mitochondrial ROS production [33,34].

## 3. Experimental Section

### 3.1. Chemicals and Materials

Actinomycin D, antimycin A, dimethylsulfoxide, monodansylcadaverine, and wortmannin were purchased from Sigma-Aldrich (St. Louis, MO, USA). Dulbecco’s modified Eagle’s medium (DMEM) and fetal bovine serum (FBS) were obtained from Invitrogen (Grand Island, NY, USA). The MitoSOX and TUNEL kits were obtained from Invitrogen (Eugene, OR, USA). All of the other reagents were analytical-grade.

### 3.2. Isolation of (−)-Elatol from L. dendroidea

The halogenated sesquiterpene (−)-elatol was isolated from specimens of the red macroalga *L. dendroidea* collected by hand during low tide in the midlittoral zone on the rocky coast of Cabo Frio Island (22°59′ S, 42°59′ W), Rio de Janeiro State, Brazil. The seaweed was transported to the laboratory between moist paper sheets inside individual plastic bags that were placed in coolers. These specimens were dried in the dark at room temperature to avoid photolysis and thermal degradation. Dr. Mutue Toyota Fujii identified the specimens of *L. dendroidea* used in this study, and voucher specimens were deposited in the herbaria SP, Instituto de Botânica, São Paulo State, Brazil (SP no. 399789).

The freeze-dried algal material (300 g) yielded 50 mg (−)-elatol through successive and exhaustively extraction in *n*-hexane at room temperature for 15 days. The solvent was eliminated in a rotary evaporator at low temperature (<50 °C), yielding 3.64 g of a dark green extract that contained the sesquiterpene (−)-elatol, which was detected as a blue spot on TLC plates after spraying with a solution of ceric sulfate and sulfuric acid (2.1 g Ce_2_[SO_4_]_3_·4H_2_O, 21 mL H_2_SO_4_, and 300 mL H_2_O), followed by heating at 100 °C for 3 min. An aliquot of the extract (0.35 g) was subjected to preparative thin-layer chromatography (PTLC; Merck; silica gel 60 F_254_, 20 × 20 cm, mobile phase: *n*-hexane:ethyl acetate [8:2]), to yield a yellowish oil (50 mg) that was identified as the sesquiterpene (−)-elatol. The purity was confirmed by TLC (*R_f_* = 0.45) using *n*-hexane:AcOEt (8:2) as the mobile phase and ^1^H-NMR spectroscopy (300 MHz), and comparisons were made with the literature [35].

(−)-Elatol stock solutions (1 mg/mL) were prepared in dimethylsulfoxide (DMSO). All of the groups (including controls) were tested at final concentrations of less than 1% DMSO, which has no effect on amastigotes (data not shown). The concentrations of (−)-elatol used in the assays were equal to and above 50% inhibitory concentration (IC_50_) value [10].

### 3.3. Parasites and Cells

All of the experiments were performed using the Y strain of *T. cruzi*. Amastigote forms were obtained from the supernatants of previously infected monolayers of LLCMK_2_ cells (epithelial cells of monkey kidney [*Macaca mulatta*]; CCL-7; American Type Culture Collection, Rockville, MD, USA) in DMEM supplemented with 2 mM L-glutamine, 10% FBS, and 50 mg/L gentamicin and buffered with sodium bicarbonate in a 5% CO_2_ air mixture at 37 °C.

### 3.4. Fluorimetric Detection of Mitochondrion-Derived O_2_^•−^

The mitochondrial production of O_2_^•−^ was evaluated during exposure of the amastigotes to 3, 15 and 30 μM of (−)-elatol using the fluorescent O_2_^•−^-sensitive, mitochondrial-targeted probe MitoSOX (3,8-phenanthridinediamine,5-[6-triphenylphosphoniumhexyl]-5,6-dihydro-6-phenyl). Amastigotes (2 × 10^7^ cells/mL) were preloaded with 5 μM MitoSOX for 10 min at room temperature and then washed with Krebs-Henseleit buffer (pH 7.3) that contained 15 mM NaHCO_3_, 5 mM KCl, 120 mM NaCl, 0.7 mM Na_2_HPO_4_, and 1.5 mM NaH_2_PO_4_ before the assays. Loaded cells were exposed to different concentrations of (–)-elatol. After different times (0–3 h), fluorescence was measured in a fluorescence microplate reader (Victor X3, PerkinElmer) at λ_ex_ = 510 nm and λ_em_ = 580 nm. Oxidized MitoSOX becomes highly fluorescent upon binding to nucleic acids. In some of the experiments, the cells were exposed to 10 μM antimycin A, which is known to induce mitochondrial O_2_^•−^ production [36].

### 3.5. Evaluation of DNA Fragmentation

DNA fragmentation was evaluated *in situ* using terminal deoxynucleotide transferase dUTP nick-end labeling (TUNEL). Amastigote forms (1 × 10^7^ cells/mL) were treated with 1.5 and 3 μM (−)-elatol for 24 h at 37 °C after the cells were subjected to the TUNEL assay according to the manufacturer’s instructions. The compound actinomycin D (20 mM) was used as a positive control. Cells that undergo DNA double-strand ruptures should fluoresce brightly, unlike untreated cells. Fluorescence was observed in a fluorescence microscope Olympus BX51 (Olympus^®^, Tokyo, Japan) at λ_ex_ = 495 nm and λ_em_ = 519 nm, and images were captured with a UC30 camera (Olympus^®^).

### 3.6. Evaluation of Autophagic Compartments

Autophagic compartments were evaluated using monodansylcadaverine labeling [37]. Amastigote forms (1 × 10^7^ cells/mL) were treated with 1.5 and 3 μM (−)-elatol for 24 h at 37 °C. The cells were then incubated with 0.05 mM monodansylcadaverine in phosphate-buffered saline (PBS) for 15 min at 37 °C. After incubation, the cells were washed in PBS two times. Monodansylcadaverine staining was analyzed using a fluorescence microscope Olympus BX51 (Olympus^®^) at λ_ex_ = 380 nm and λ_em_ = 525 nm, and images were captured with a UC30 camera (Olympus^®^). In some of the experiments, the cells were pretreated with 500 nM wortmannin before the induction of autophagy. The compound is a potent phosphatidylinositol 3-kinase (PI3K) inhibitor, an enzyme that is involved in autophagy regulation [38].

### 3.7. Statistical Analysis

The data shown in the graphs are expressed as the mean ± standard deviation of at least three independent experiments. The data were analyzed using two-way analysis of variance (ANOVA), and significant differences among means were identified using the Tukey *post hoc* test. Values of *p* ≤ 0.05 were considered statistically significant. The statistical analyses were performed using Statistica software.

## 4. Conclusions

In summary, the present study provided further insights into the effects of (–)-elatol on amastigote forms of *T. cruzi* in an attempt to find new and specific therapies for Chagas’ disease. (–)-Elatol might be an effective compound for further *in vivo* analysis and may be a prototypical compound for the development of synthetic derivatives that may be used to treat this infection that affects millions of people in Latin America.
